# FAT FLUX: enzymes, regulators, and pathophysiology of intracellular lipolysis

**DOI:** 10.15252/emmm.201404846

**Published:** 2015-01-21

**Authors:** Rudolf Zechner

**Affiliations:** Institute of Molecular Biosciences, University of GrazGraz, Austria

## Abstract

The great 19^th^ century French physiologist Claude Bernard reasoned “Man can learn nothing except by going from the known to the unknown”. This premise is particularly applicable to the progression of discoveries made in the field of fat metabolism since Bernard's time. Beginning with his groundbreaking discovery of fat digestion (later termed “lipolysis”) in 1848, research addressing the basic processes of cellular storage and mobilization of fat has steadily advanced. Even after 150 years of research dedicated to lipolysis, exciting new principles have continued to emerge in the last 10 years. This Perspective summarizes these recent landmark discoveries in the field and emphasizes their relevance for the pathogenesis of extremely prevalent diseases such as obesity, heart disease, and cancer.

## A brief history of fat and fat catabolism

Research in my laboratory revolves around fatty acids (FAs), fundamental biomolecules in essentially all organisms. Their importance is due to their role as constituents of all lipid classes including lipids that form biological membranes, and the fact that FAs are the most efficient “fuel” for energy production in most eukaryotes. Indeed, a constant supply of FAs is required to maintain organism homeostasis. However, when present in high concentrations, FAs exert toxic effects by perturbing the cellular acid/base balance, disrupting membrane structures, and generating harmful (“lipotoxic”) compounds, which cause cell and tissue dysfunction. To enable efficient storage without these harmful side effects, FAs are esterified to glycerol. The resulting triglycerides (TGs) are referred to as fat. TGs are relatively inert and can be efficiently deposited in lipid droplets (LDs) in the cytoplasm of essentially all cell types. The most efficient depot of TGs in mammals is white adipose tissue (WAT). As early as at the beginning of the 19th century, the French chemists Chevreul and Berthelot had elucidated the composition of TGs in animal and plant “fats” and “oils”, respectively. As mentioned above, in 1848 Claude Bernard was the first to discover lipolysis as the enzymatic process that “saponificates” (hydrolyzes) TGs. The general biological importance of lipolysis was recognized once it was understood that TGs are unable to cross cell membranes. Accordingly, all nutritional fat must be hydrolyzed before it can be absorbed in the gut and later deposited in adipose and/or non-adipose tissues. Conversely, when fat is mobilized again from adipose tissue to supply other tissues such as heart, muscle, or liver with energy, TGs must be degraded before FAs and glycerol can be released.

## The biochemistry of lipolysis

Although the need for TG hydrolysis to release FAs from WAT was recognized in the early 20^th^ century, it took more than 50 years to identify some of the participating enzymes. In 1964, D. Steinberg and colleagues published a landmark paper showing that hormone-sensitive lipase (HSL), in conjunction with monoglyceride lipase (MGL), hydrolyzes TGs to generate FAs and glycerol (Vaughan *et al*, 1964). The role of HSL as the “rate-limiting” enzyme in WAT lipolysis remained unchallenged for decades until several groups including my laboratory inactivated the gene coding for HSL in mice. Against all expectations, these animals exhibited relatively normal TG catabolism and instead accumulated marked amounts of diglycerides (DGs) in many tissues. This finding identified a more important function for HSL in hydrolyzing DGs rather than TGs and fueled predictions that alternative lipase(s) facilitate(s) the cellular catabolism of fat.

In 2004, three groups simultaneously reported the discovery of a potential “alternative” TG hydrolase very likely to assume a role in fat catabolism in WAT. We named this enzyme adipose triglyceride lipase (ATGL) (Zimmermann *et al*, 2004) and demonstrated that its overexpression or inhibition strongly affected the lipolytic breakdown of cellular TG stores. ATGL belongs to a protein family containing a patatin-domain first identified in the abundant potato tuber protein patatin. Many members of this protein family exhibit lipid ester hydrolase activity via an unusual catalytic dyad in the active site of the enzyme.

Ultimate proof for the essential role of ATGL as TG lipase *in vivo* came from the enlightening phenotype of ATGL-knockout mice (Haemmerle *et al*, 2006). Unlike HSL-deficient mice, these animals accumulated TGs in multiple tissues including liver, heart, skeletal muscle, kidney, testis, and macrophages and developed obesity when fed a normal diet. The most severe defect was observed in cardiac muscle where ATGL deficiency led to massive TG accumulation, mitochondrial dysfunction, and cardiomyopathy causing premature death 3–4 months after birth. This extreme cardiac phenotype highlighted a crucial role of lipolysis also in a non-adipose tissue with high FA turnover.

We subsequently found that full ATGL enzymatic activity requires a co-activator named comparative gene identification-58 (CGI-58) or A/B hydrolase domain containing-5 (ABHD5) (Lass *et al*, 2006). In WAT, CGI-58 binds to the LD structural protein perilipin-1. Upon hormonal stimulation by β-adrenergic agonists, CGI-58 dissociates from perilipin-1 and interacts with ATGL increasing enzyme activity up to 20-fold. Mice lacking CGI-58 develop a similar lipid accumulation phenotype as ATGL-deficient mice, but, in addition, develop a severe epidermal skin defect where desiccation due to massive trans-epidermal water loss results in perinatal death within 12–16 h after birth (Radner *et al*, 2010). The lack of skin pathology in ATGL-deficient mice clearly indicated that CGI-58 has an ATGL independent function in the skin and possibly other tissues. Several alternative functions for CGI-58 including enzymatic activity as an acyl-transferase have been proposed and await corroboration. More recently, Liu and colleagues reported the discovery of a specific peptide repressor of ATGL named G0/G1 switch protein-2 (G0S2) (Yang *et al*, 2010). This factor interacts with the patatin-domain of ATGL and inhibits the enzyme by a non-competitive mechanism. Furthermore, numerous other factors and hormones have been reported to indirectly regulate ATGL-mediated lipolysis.

## Biochemical basis for an inherited human disease

The discovery of ATGL and its activation by CGI-58 provided a mechanistic explanation for the pathogenesis of neutral lipid storage disease (NLSD) in humans. NLSD patients are characterized by the systemic accumulation of fat in multiple tissues. Mutations in CGI-58 cause NLSD with ichthyosis (NLSDI, also named Chanarin–Dorfman syndrome; Lefevre *et al*, 2001). The crucial function of CGI-58 in ATGL-catalyzed lipolysis provided a biochemical mechanism for the excessive lipid storage observed in these patients. Later a second group of NLSD patients was identified with mutations in the gene coding for ATGL (*PNPLA2*) (Fischer *et al*, 2007). Similarly as observed in ATGL-null mice, these patients exhibited severe fat accumulation in skeletal and cardiac muscle often leading to a potentially lethal cardiomyopathy (Hirano *et al*, 2008). The pronounced muscle pathology in patients affected with mutations in ATGL led to its designation as NLSD with myopathy (NLSDM).

Taken together, the symptoms observed in patients that lack ATGL or CGI-58 strongly resemble the phenotype of the respective knockout mice and highlight the importance of lipolysis in adipose and non-adipose tissues. The distinct outcomes depending on whether patients or mice lack the enzyme or its co-activator suggest important additional, possibly lipolysis-independent functions. This implied broader range of function is an area of current research interest.

## Lipolysis, lipid signaling, and energy metabolism

**Figure 1:**
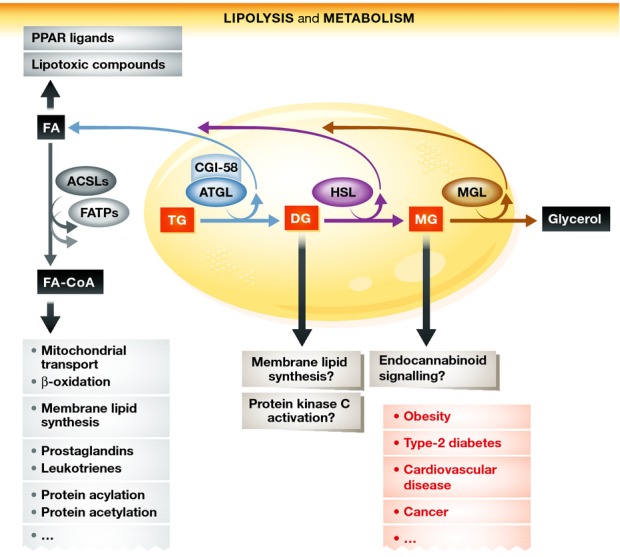
Lipolysis and its potential effects on metabolic and signaling pathways Lipolysis of TGs occurs in lipid droplets (yellow) via the enzymatic activities of ATGL, HSL, and MGL. Lipolysis generates FAs and glycerol. FAs and the lipolytic intermediates DGs and MGs are important signaling lipids or precursors for signaling lipids. Additionally, they are major energy substrates and precursors for the synthesis of membrane lipids. Alterations in lipolysis may alter these pathways and contribute to the development of the metabolic diseases listed in red. ACSL, acyl-CoA synthetase; ATGL, adipose triglyceride lipase; CGI-58, comparative gene identification-58; CoA, coenzyme A; DG, diglyceride; FA, fatty acid; FATP, FA transport protein; HSL, hormone-sensitive lipase; MG, monoglyceride; MGL, MG lipase; TG, triglyceride.

The complex disease profile observed in patients and mice lacking either ATGL or CGI-58 also suggested that defects in lipolysis not only regulate fat storage in WAT but also affect energy homeostasis more generally by interfering with cellular signaling pathways. Figure1 summarizes the lipolytic pathway as well as the intermediates and end products that may either directly or indirectly intervene with cellular signaling. Several groups, including ours, have shown that lipolysis is required for functional peroxisome proliferator-activated receptor (PPAR) signaling (Haemmerle *et al*, 2011). For example, defective lipolysis in cardiac myocytes of mice leads to a marked decrease in the transcription of PPARα target genes resulting in lower abundance of mitochondria and impaired respiratory function. This loss of oxidative power leads to the cardiomyopathy and early death seen in ATGL-knockout mice. The finding that pharmacological treatment of ATGL-deficient mice with PPARα agonists helped to lessen the cardiac phenotype supported the hypothesis (which remains to be formally proven) that lipolysis generates FAs, which act as PPAR ligands and thereby transactivate target gene transcription. Recent evidence further suggests that ATGL-mediated lipolysis also affects PPARδ and PPARγ signaling. HSL deficiency in mice or humans also leads to decreased PPARγ activity and reduced lipogenesis and lipid synthesis in WAT (Albert *et al*, 2014).

Another example of how lipolysis affects cellular signaling pathways was provided by the demonstration that MGL interferes with endocannabinoid signaling, due to its ability to control the cellular concentration of the main endocannabinoid, 2-arachidonylglycerol (2-AG) (Schlosburg *et al*, 2010).

Lipolysis also strongly affects insulin signaling. Despite pronounced TG accumulation, ATGL-deficient mouse tissues are highly insulin sensitive and glucose tolerant. High insulin sensitivity and increased glucose uptake in muscle, liver, and adipose tissue is observed during states of low plasma insulin concentrations and decreased glucose-stimulated insulin secretion from ATGL-deficient pancreatic β-cells (Tang *et al*, 2013). Interestingly, heterozygous or homozygous HSL deficiency in mice is also associated with high insulin sensitivity (Girousse *et al*, 2013), while humans with HSL deficiency become insulin resistant (Albert *et al*, 2014). Although HSL-deficient mice and humans accumulate DGs in adipose tissue, activation of protein kinase (PKC) isoforms and the subsequent inactivation of the insulin-signaling pathway are not observed. This may be explained by the fact that the ATGL/CGI-58 reaction does not generate sn-1,2 DGs, the DG isoform that activates PKC.

We have also recently learned that lipolysis and lipolytic enzymes contribute to the regulation of growth signaling, cancer development, and the pathogenesis of cancer-associated cachexia (CAC). The involved mechanisms are mostly unknown and the focus of exciting current research. For example, B. Cravatt's group demonstrated that MGL promotes cancer growth in mice and concluded that cancer cells utilize a lipolytic enzyme to generate protumorigenic signals (Nomura *et al*, 2010). Most recently, L. Yu and colleagues showed that CGI-58 acts as a tumor suppressor in a colon cancer mouse model (Ou *et al*, 2014). Although the underlying mechanisms require detailed characterization, these findings provide compelling evidence for an important role of lipolysis in cancer development and promotion. Results from our laboratory demonstrated that lipolysis is also involved in the pathogenesis of CAC. Cachexia is a devastating condition of uncontrolled loss of adipose and muscle mass commonly observed in patients with cancer and other chronic diseases. CAC is associated with a very poor prognosis for survival. Encouragingly, in a recent study, we were able to show that in the absence of ATGL (and HSL), the development of CAC can be delayed in certain mouse models of cancer (Das *et al*, 2011).

## Perspective

During the past 10 years, important discoveries have helped define the enzymology and regulation of lipolysis. Newly discovered enzymes and regulators are providing a more complete picture of the lipolytic pathway, and a plethora of agonists and antagonists has been described that regulates the lipolytic degradation of fat. In contrast, our understanding of how lipolysis conversely affects gene transcription, post-translational protein modifications, or cell signaling is much less developed. Only very recently has the impact of lipolysis on PPAR signaling, insulin sensitivity, or cell growth been revealed. We now need to elucidate the detailed mechanisms behind these processes. These insights will eventually help define whether pharmacological intervention designed to interfere with the lipolytic pathway can be effective in the treatment of insulin resistance and type 2 diabetes, cancer cell growth, or CAC.
